# Investigation of native and aggregated therapeutic proteins in human plasma with asymmetrical flow field-flow fractionation and mass spectrometry

**DOI:** 10.1007/s00216-022-04355-2

**Published:** 2022-10-05

**Authors:** Ingrid Ramm, Mats Leeman, Herje Schagerlöf, Ileana Rodríguez León, Alejandra Castro, Lars Nilsson

**Affiliations:** 1 Department of Food Technology, Engineering and Nutrition, Lund University, 221 00 Lund, Sweden; 2 SOLVE Research and Consultancy AB, Medicon village, 223 81 Lund, Sweden; 3 Present Address: Occupational and Environmental Medicine, Skåne University Hospital, Medicon village, 223 81 Lund, Sweden; 4 Department of Chemical Engineering, Lund University, 221 00 Lund, Sweden; 5 Present Address: Global Research Technology, Novo Nordisk A/S, 2760 Måløv, Denmark; 6 Analytical Development – Methods and Characterization, Product Development and Drug Delivery, Global Pharmaceutical R&D, Ferring Pharmaceuticals A/S, Amager Strandvej 405, 2770 Kastrup, Denmark

**Keywords:** Asymmetrical flow field-flow fractionation, Liquid chromatography–mass spectrometry, Plasma, Antibody, Aggregate, Detection

## Abstract

Physiochemical degradation of therapeutic proteins in vivo during plasma circulation after administration can have a detrimental effect on their efficacy and safety profile. During drug product development, in vivo animal studies are necessary to explore in vivo protein behaviour. However, these studies are very demanding and expensive, and the industry is working to decrease the number of in vivo studies. Consequently, there is considerable interest in the development of methods to pre-screen the behaviour of therapeutic proteins in vivo using in vitro analysis. In this work, asymmetrical flow field-flow fractionation (AF4) and liquid chromatography–mass spectrometry (LC-MS) were combined to develop a novel analytical methodology for predicting the behaviour of therapeutic proteins in vivo. The method was tested with two proteins, a monoclonal antibody and a serum albumin binding affibody. After incubation of the proteins in plasma, the method was successfully used to investigate and quantify serum albumin binding, analyse changes in monoclonal antibody size, and identify and quantify monoclonal antibody aggregates.

**Graphical abstract:**

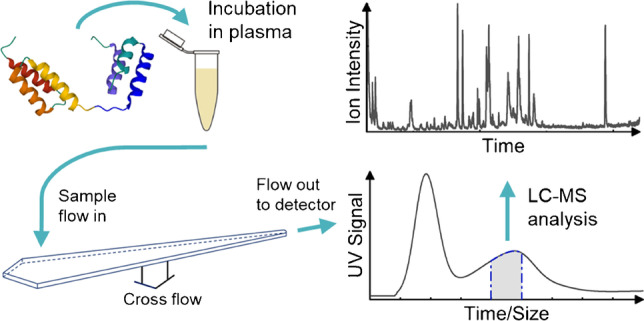

**Supplementary Information:**

The online version contains supplementary material available at 10.1007/s00216-022-04355-2.

## Introduction

Because of the improved understanding of the diverse roles of proteins in human physiological processes, therapeutic proteins are becoming a key factor in modern medicine. These proteins can target and regulate highly specific and complicated biological processes and can be used in the treatment of many different diseases. As of 2021, out of the ten most widely sold drugs globally, seven were therapeutic proteins. Five of these drugs are antibodies and two are fusion proteins [1]. Other examples of proteins used as therapeutic proteins are blood/growth factors, enzymes, hormones, and tumour necrosis factors [2].

Compared with traditional small-molecule drugs, therapeutic proteins are large, complex, and sensitive, having both a secondary and a tertiary structure that need to be maintained to preserve their function. Therapeutic proteins can degrade both chemically and physically, leading to loss of functionality and adverse immunogenic effects during therapy [3–7]. Therefore, it is necessary to examine the rate and extent of degradation of the therapeutic proteins in their formulations, which are developed to minimise the risk of degradation [1, 8]. Most therapeutic proteins are administered either through subcutaneous injection or through intravenous infusion directly into the systemic circulation [1, 8, 9]. After subcutaneous administration, the therapeutic proteins diffuse or are transported into the blood circulation [10], which is a complex matrix comprising a plasma phase and a cellular phase. Blood differs from the formulation of therapeutic proteins in composition and often also in its physiochemical and physical properties, such as pH and ionic strength. Hence, the stability of the protein may differ between the formulation and blood. Therefore, it is also necessary to analyse the stability and behaviour of the protein in vivo, including studies of immunogenicity, bioavailability, pharmacokinetics, and toxicokinetics. In vivo studies are expensive and demanding, and require animal and patient studies; hence, the pharmaceutical industry is working to decrease the number of in vivo studies. It would be of high interest to develop methods that can be utilised to pre-screen the behaviour of therapeutic proteins in vivo by using in vitro analyses. In vitro methods used to predict the aggregation of therapeutic proteins in vivo, depending on their formulation, have been shown to provide valuable information, and support the usability of such prediction methods [11].

Bioanalytical techniques must be able to determine the properties of the therapeutic proteins in the very complex matrix of blood. Blood plasma constitutes 55% of the blood volume [12]. It contains proteins (~60–80 mg/mL), salts, lipids (~6 mg/mL), sugars (~0.8 mg/mL), nutrients, waste products, and regulatory substances [2, 12, 13]. The most abundant proteins are albumins (57–60 wt%), immunoglobulins (~40 wt%), and fibrinogens (4 wt%) [12, 13]. These three protein fractions together constitute more than 90 wt% of the total protein content. However, the number of different proteins in plasma can exceed 10,000 [14, 15]. In serum, fibrinogen and clotting factors have been removed by blood clotting before centrifugation for removing the cellular phase [16].

Asymmetrical field-flow fractionation (AF4) is an analytical technique that separates analytes depending on size [17, 18]. The separation is achieved by applying a flow along a separation channel that is devoid of a stationary phase. A perpendicular crossflow is applied which transports the analytes towards the bottom of the channel (accumulation wall), where the crossflow exits through an ultrafiltration membrane. The cut-off of the membrane can vary depending on the sample being analysed. The crossflow-induced transport to the accumulation wall is counteracted through the diffusion of analytes. At steady state, a concentration profile is established which depends on the diffusion coefficient (hydrodynamic size) and decays exponentially away from the accumulation wall. As the longitudinal flow has a parabolic flow profile, analytes that have a longer average distance (higher diffusion coefficient) from the accumulation wall will experience higher flow velocity along the separation channel. In this way, size separation is achieved. AF4 can be connected to several detectors, for example, UV, differential refractive index, and multi-angle light-scattering, and provide valuable information on composition and sample properties in fractions eluting from the AF4 channel (concentration, size, molecular weight, etc.). AF4 has increasingly been used for the characterisation and separation of pharmaceutical compounds and biomacromolecules [19–21]. AF4 has also been used to analyse different colloids, such as lipoproteins, in plasma [22–24] and was used in a study by Madörin et al. to analyse the distribution of a small-molecule drug in human plasma [25]. Analysis of whole blood, plasma, and serum with AF4 can be performed with conditions very similar to the physiological conditions in vivo, i.e. maintaining physiological salinity and pH and avoiding surfactants and organic solvents [13]. Moreover, since the method lacks a stationary phase, shear forces and the internal surface area are low compared with size exclusion chromatography (SEC), generating lower stress on the samples. The surface area of an SEC column is, depending on the specific column, approximately 10^3^–10^4^ greater than that of AF4, and the shear forces are in the order of 10^3^ higher in SEC. To reduce interactions between the sample and the stationary phase in SEC, it is also often necessary to apply a relatively high concentration of salt in the mobile phase or use other additives [26, 27]. Hence, the relevance of such analyses can be greatly limited when compared with a native or formulated environment of a protein.

In two previous studies by Leeman et al., it was shown that AF4 can be used to characterise therapeutic proteins (antibodies) in serum and plasma [13, 26]. Since the native plasma proteins and the therapeutic antibodies have overlapping hydrodynamic sizes, the occurrence of co-elution is inevitable, and fractionation of single proteins is thus not possible. Consequently, it was necessary to add a method for the selective identification and quantitation of the therapeutic proteins of interest. For this, fluorescent labelling [13] and surface plasmon resonance (SPR) [26] were used. However, both approaches can suffer from various drawbacks. The labelling of a protein will imply a modification of the chemical structure, which in turn may lead to changes in the physiochemical properties of the protein. To obtain selective detection with SPR, it is necessary to immobilise specific ligands to the SPR metal surface. The immobilisation of the ligand can become somewhat cumbersome and may require optimisation. Another potential drawback is the question of how selective and quantitative the SPR response becomes for protein oligomers, complexes, and aggregates [13, 26]. Hence, it is of interest to investigate the application of other sensitive techniques for the selective analysis of the fractions eluting from the AF4 channel, and one choice is the use of liquid chromatography–mass spectrometry (LC-MS). Field-flow fractionation has previously been combined with LC-MS to analyse native proteins, lipoproteins, exosomes, and whole bacteria [28–33].

The most common bioanalytical techniques for quantitative and qualitative analysis used to support the drug development of therapeutic proteins are ligand binding assays, such as enzyme-linked immunosorbent assay (ELISA) and SPR. The detection levels of these methods are often lower than for LC-MS. However, the use of LC-MS has increased over the years because of its high selectivity, specificity, precision, and accuracy. LC-MS can also be easier to implement for different protein systems since it is not necessary to develop a new assay for each protein. This makes the technique suitable for analyses of complex mixtures such as plasma and serum. LC-MS analysis also reduces problems seen in ligand binding assays such as non-specific binding and cross-reactivity [2, 34, 35]. However, endogenous compounds can interfere with the LC-MS analysis. Therefore, to achieve high specificity and accuracy, and to be able to detect low levels of the analytes of interest, one or more purification steps are needed before the LC-MS analysis. The combination of AF4 with LC-MS can provide information on the size of analytes eluting from the AF4 separation channel, as well as identifying and quantifying specific analytes. During the AF4 fractionation, smaller species (with lower molecular weight [M_W_] than the cut-off of the ultrafiltration membrane) will be removed. AF4 can thus also serve as a purification step to remove species that could potentially interfere with the LC-MS analyses.

The aim of this study was to develop a novel in vitro methodology to provide insight into the behaviour of therapeutic proteins in vivo. The method used is a combination of AF4 and LC-MS. The separation with AF4 gives valuable information on size which, for example, can indicate binding, oligomerisation, and aggregation. Two different proteins are utilised, a monoclonal antibody and a serum albumin binding affibody. The proteins were chosen due to their high relevance in medical and biotechnological applications. Monoclonal antibodies constitute the most widely sold class of therapeutic proteins [1], and affibodies are a class of engineered proteins of increasing interest [36, 37]. The purpose of this work is to demonstrate that after incubation in plasma, (1) serum albumin binding can be investigated and quantified, (2) changes in monoclonal antibody size (oligomerisation and/or interaction with blood components) can be analysed, and (3) monoclonal antibody aggregates can be identified and quantified.

## Material and methods

### Materials and reagents

The affibody GA-Z, in a buffer with 25 mM sodium phosphate, 125 mM NaCl, pH 7.0, and a stock concentration of 90 mg/mL, was supplied by Swedish Orphan Biovitrum AB (Stockholm, Sweden). The protein was stored at −80 °C before use. The molecular weight and isoelectric point of GA-Z are 11,865 Da and 4.5, respectively [38]. The monoclonal antibody trastuzumab was supplied by Bernt Nilsson (Lund University, Sweden) [39, 40] at a stock concentration of 8.4 mg/mL in a buffer with 25 mM sodium phosphate buffer, 25 mM sodium acetate, and 120 mM NaCl at pH 6.2. Trastuzumab has a molecular weight of 148,300 Da and an isoelectric point of 8.7 [41]. Tris hydrochloride (molecular biology grade, ≥99%), 1,4-dithiothreitol (Ellman′s reagent, ≥97%), iodoacetamide (BioUltra, ≥99%), sodium deoxycholate (≥97%), trypsin porcine (MS grade), plasma (human, P9523), and trifluoroacetic acid (HPLC grade, > 99%) were purchased from Sigma-Aldrich (St. Louis, MO, USA). Formic acid (LC-MS grade), sodium chloride (NaCl, ≥98%), disodium hydrogen phosphate dihydrate (Na_2_HPO_4_•2H_2_O, 101%), potassium dihydrogen phosphate (H_2_KPO_4_, 101%), potassium chloride (KCl, 99.9%), sodium hydroxide (NaOH), and sodium azide (NaN_3_) were purchased from VWR (Radnor, PA, USA). Formic acid (LC-MS grade) was purchased from Fisher Chemical (NH, USA). Phosphoric acid (HPO_3_, pro-analytical quality) was purchased from Merck Millipore (Burlington, MA, USA). Acetonitrile (LC-MS grade, 99.9%) was purchased from Honeywell (Charlotte, NC, USA). Water used for samples, buffers, carrier liquids, and mobile phases was of Milli-Q grade (Merck Millipore).

### Aggregation of trastuzumab

Trastuzumab was aggregated by lowering the pH to 2.5 with 1 M phosphoric acid (Merck Millipore). During the addition, the sample with trastuzumab was mixed carefully. The pH was then increased to 6.2 by adding 0.5 M sodium hydroxide (VWR).

### Fractionation with asymmetrical flow field-flow fractionation

Plasma and serum samples were fractionated and characterised using asymmetrical flow field-flow fractionation (AF4). GA-Z, trastuzumab, or aggregated trastuzumab were incubated in plasma for 30 min at 20 °C, in which 100 μL plasma was spiked with 90 μg of GA-Z or trastuzumab. The incubated samples were diluted tenfold with phosphate-buffered saline (PBS; pH 7.4), before the injection on AF4. AF4 was performed with a short trapezoidal separation channel (Wyatt Technology, Santa Barbara, CA, USA) having a tip-to-tip length of 17.4 cm, an inlet width of 2.15, and an outlet width of 0.3 cm. The spacer was 350 μm thick and the membrane was of regenerated cellulose with a molecular weight cut-off of 10 kDa (Merck Millipore). For the AF4 analysis, an Eclipse 2 system (Wyatt Technology) equipped with an Agilent 1100 HPLC pump and autosampler (Agilent Technologies, Santa Clara, CA, USA) was used. The system was connected to a 1100 UV detector (Agilent Technologies) and the UV detector was operating at 280 nm. PBS buffer, at pH 7.4 with 160 mM sodium ions, 140 mM chloride ions, 5 mM potassium ions, 12 mM phosphate ions, 0.02% wt sodium azide (VWR), in Milli-Q water was used as the carrier liquid. The detector flow was 0.5 mL/min, and the crossflow was constant at 2 mL/min for 16 min and then exponentially decreased to 0.15 mL/min over 16 min, according to Fig. S1 in the Supplementary Information. The relaxation time was 5 min. The injection flow was 0.2 mL/min, the injection time was 2 min, and the injection volume was 10 μL. The samples had a protein concentration of ~7 mg/mL, corresponding to an injected mass of approximately 70 μg. A change in injected mass (14 μg or 70 μg) did not change the retention time of the sample, indicating that the channel was not overloaded. Analysis was performed in triplicate for all samples, and each sample was analysed directly after the incubation time. The experiments were performed at 20 °C in a temperature-regulated room. Data analysis was performed with ASTRA 6.1 software (Wyatt Technology). During the run, fractions were collected with a time interval of 1 min. The fractions were stored at −80 °C and then freeze-dried before further analysis.

### Determination of hydrodynamic radii

The hydrodynamic radii of the eluted samples were calculated using protein standards. The standards were used to create a calibration curve of elution time vs. hydrodynamic radius. The fractograms of the protein standards and the determination of the hydrodynamic radii are shown in Fig. S2 in the Supplementary Information.

###  Tryptic digestion

To be able to analyse the proteins in the AF4 fractions using LC-MS, tryptic digestion was performed. The AF4 fractions were dissolved in 100 μL of 1 wt% sodium deoxycholate (Sigma-Aldrich) in Milli-Q water and incubated for 10 min at 37 °C to denature the proteins. Samples were reduced by adding 2 μL of 0.5 M dithiothreitol (Sigma-Aldrich) in water to a final concentration of ~10 mM and incubating the samples for 60 min at 37 °C. Free SH-groups were alkylated by adding 2 μL of 1 M sodium iodoacetamide (Sigma-Aldrich) in water to a final concentration of ~20 mM and incubating the samples for 40 min at room temperature in the dark. Before the tryptic digestion the samples were diluted with 100 μL of 50 mM Tris-HCl, pH 7.4 (Sigma-Aldrich) to lower the concentration of sodium deoxycholate from 1 wt% to 0.5 wt%. 0.4 μg of trypsin (Sigma-Aldrich) was added to a weight ratio of 1:25–1:50 (trypsin/protein) and the samples were incubated for 4 h at 37 °C during the digestion. To terminate the digestion and to precipitate sodium deoxycholate, 2 μL of formic acid (VWR) was added to a obtain final concentration of > 0.5% v/v. Precipitated sodium deoxycholate was removed with stepwise centrifugation (15 min at 30,000g followed by 30 min at 30,000g) and the supernatant was transferred to new vials. Samples containing GA-Z were directly analysed with LC-MS. Samples with trastuzumab were freeze-dried and redissolved in a lower volume to concentrate the samples and then directly analysed with LC-MS.

### Liquid chromatography–mass spectrometry

The trypsin-digested AF4 fractions were analysed with LC-MS to detect and quantify the amount of signature peptides of GA-Z or trastuzumab. The analysis was performed with an Agilent 1260 Infinity II system (Agilent Technologies) connected to an Agilent 6545 quadrupole time-of-flight (Q-TOF) LC-MS system (Agilent Technologies). The Agilent 1260 Infinity II system was equipped with an Agilent 1260 Infinity II autosampler, an Agilent Infinity 1260 II pump, and an XBridge BEH C18 XP column (130Å, 2.5 μm, 3 × 150 mm, Waters, Milford, MA, USA). A gradient was run with mobile phase A: 0.1% formic acid (Fisher Chemical) in Milli-Q water, and mobile phase B: 0.1% formic acid (Fisher Chemical) in acetonitrile (Honeywell). Mobile phase B was constant at 5% during the period at 0–3 min, increased 5–55% during the period at 3–38 min, and increased 55–95% in the period at 38–42 min. The flow was 0.3 mL/min, the column oven temperature was 35 °C, and the injection volume was 5 μL for samples with GA-Z and 10 μL for samples with trastuzumab. UV signal data were collected at wavelengths of 220 and 280 nm. During the run, the samples were kept at 4 °C in the autosampler. During the period at 3–38 min, the LC flow entered the MS source. In the MS method, the mass range was set to 100–3000 m/z in positive mode, with an acquisition rate of 5 spectra s^−1^. The Dual AJS ESI was operated with a drying gas flow of 12 L/min, drying gas temperature of 350 °C, sheath gas flow of 12 L/min, sheath gas temperature of 225 °C, and a nebuliser pressure of 55 psi. The nozzle voltage was 2000 V, the capillary voltage was 4500 V, the skimmer voltage was 65 V, the fragmentor voltage was 300 V, and the octupole RF voltage was 750 V. The experimental data were analysed using MassHunter software (Agilent Technologies).

### Signature peptides

To identify and select signature peptides of GA-Z and trastuzumab, the proteins were digested at a concentration of 1 mg/mL following the method described above and analysed with LC-MS. Suitable signature peptides were selected according to the criteria described by van den Broek et al. [2], as follows: The signature peptides should ideally not contain methionine, cysteine, and tryptophan, or aspartic acid, asparagine, or glutamine neighbouring glycine, or the last two next to the N-terminal, or proline next to the carboxyl side of the cleavage site. Furthermore, the peptides should not contain two basic amino acids next to each other. Preferably, the length of the peptide should be between 8 and 15 amino acids. The signature peptides were first predicted using the ExPASy software tool [42] and the sequence of the proteins. The specific representation of the signature peptides in the human genome was confirmed using the Basic Local Alignment Search Tool (BLAST, https://blast.ncbi.nlm.nih.gov) software.

## Results and discussion

**Fig. 1:**
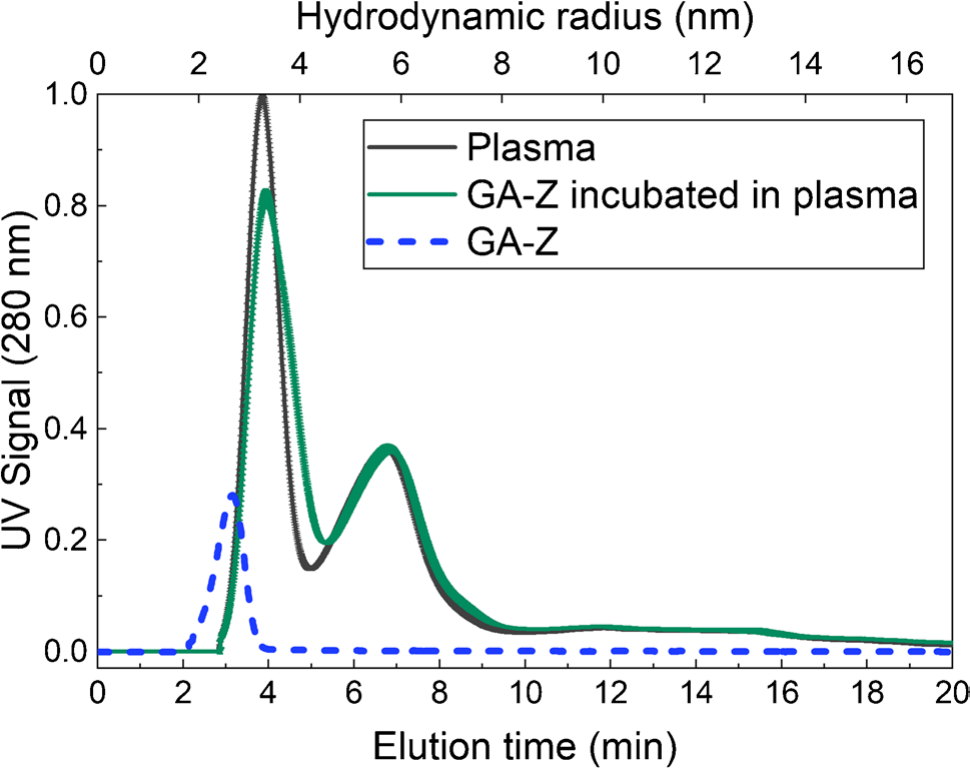
AF4-UV fractogram and hydrodynamic radius of samples with plasma (~70 μg plasma proteins) [2, 13], native GA-Z (5 μg), and GA-Z incubated in plasma (0.90 μg GA-Z + ~70 μg plasma proteins, corresponding to a molar ratio of GA-Z and HSA of approximately 1:7) for 30 min, 20 °C, in PBS buffer at pH 7.4. All analyses were performed in triplicate. The standard deviations of the fractograms are shown by the error bars. Where not visible, the error bars are overlapping with the fractogram line. All fractograms of the triplicate analyses are shown in Fig. S3 in the Supplementary Information

In this study, AF4 and LC-MS were combined to develop a method to investigate the behaviour of therapeutic proteins in plasma. An AF4 fractogram for plasma is shown in Fig. 1. The plasma fractogram contains a peak with a peak maximum at approximately 4 min dominated by human serum albumin (HSA) and a second peak at approximately 7 min dominated by immunoglobulin G (IgG), as shown by comparison with protein standards (Fig. S2, Supplementary Information), and previously by Leeman et al. [13, 26]. The fractogram also contains a population of larger analytes that start to elute at approximately 10 min, corresponding to larger components such as the larger plasma proteins, fibrinogen, IgM, and α-2-macroglobulin, or complexes of proteins [43, 44]. Because of inevitable co-elution, the two dominating peaks also contain other plasma components. The fractograms for native GA-Z and GA-Z incubated in plasma are also shown in Fig. 1. Native GA-Z shows a peak maximum at 3 min. After incubation in plasma, the native GA-Z peak is no longer present in the fractogram. Moreover, after the incubation, the HSA peak is broadened to longer elution times (larger size) and shows a decrease in intensity. GA-Z contains an HSA-binding domain and is known to bind to HSA in PBS [45]. The disappearance of the native GA-Z and the increased size of the components in the HSA peak indicate that a significant fraction of GA-Z has bound to HSA. In Fig. 1, the molar ratio of GA-Z to HSA is approximately 1:7.

**Fig. 2:**
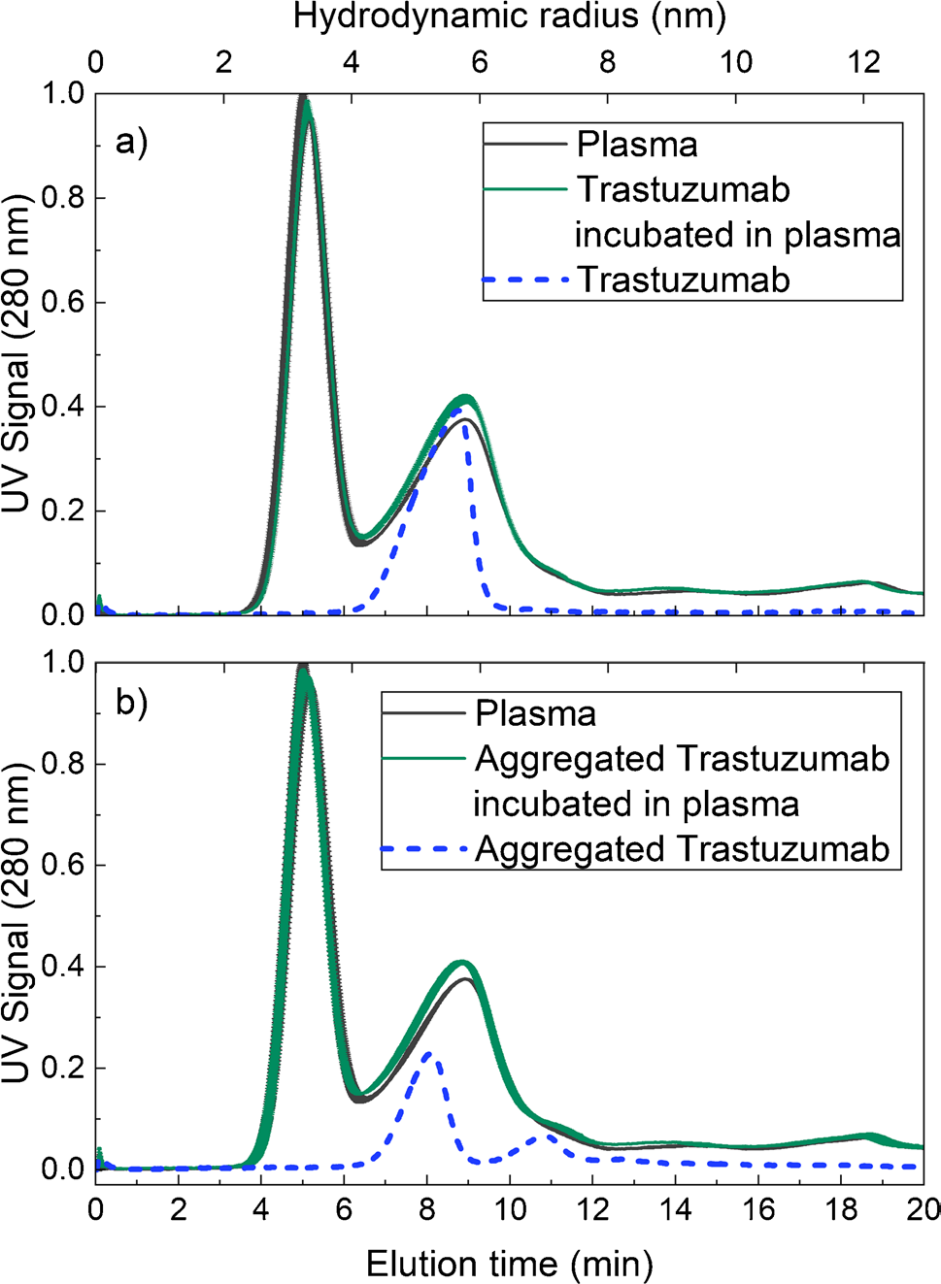
AF4-UV fractogram of samples with (**a**) plasma (~70 μg plasma proteins) [2, 13], native trastuzumab (5 μg), and trastuzumab incubated in plasma (0.82 μg trastuzumab + ~70 μg plasma proteins) for 30 min at 20 °C, and (**b**) plasma (~70 μg plasma proteins), aggregated trastuzumab (5 μg), and aggregated trastuzumab incubated in plasma (0.82 μg aggregated trastuzumab + ~70 μg plasma proteins) for 30 min at 20 °C, in PBS buffer at pH 7.4. The mass recovery of trastuzumab was determined to be 90% based on the integrated UV peak and the injected mass. Plasma, trastuzumab, and aggregated trastuzumab incubated in plasma were analysed in triplicate. The standard deviations of the fractograms are shown by the error bars. Where not visible, the error bars are overlapping with the fractogram line. The fractograms of the triplicate analyses are shown in Fig. S4 in the Supplementary Information

The AF4-UV fractograms for plasma shown in Fig. 2a, b are also dominated by an HSA and an IgG peak, eluting at approximately 5 and 9 min, respectively. Native trastuzumab and trastuzumab incubated in plasma are shown in Fig. 2a. As expected, native trastuzumab elutes at the same time as plasma IgG. After incubation, native trastuzumab co-elutes with IgG, manifested as an increase in the IgG peak. Aggregated trastuzumab and aggregated trastuzumab incubated in plasma are shown in Fig. 2b. The fractogram of aggregated trastuzumab shows a peak at approximately 8 min corresponding to native trastuzumab and a second peak at approximately 11 min corresponding to aggregated trastuzumab. The hydrodynamic radius of the aggregated trastuzumab is approximately 7 nm and is therefore ~1.5 nm larger than native trastuzumab, which has a hydrodynamic radius of approximately 5.4 nm. The fractogram of aggregated trastuzumab also shows a population with hydrodynamic radii > 8 nm eluting after 12 min, corresponding to the formation of larger aggregates. The fractogram of aggregated trastuzumab in plasma shows an increase in the IgG peak at approximately 8 min, showing co-elution of the native trastuzumab and plasma IgG. There are, however, no distinguishable peaks of the aggregated trastuzumab in the plasma fractogram, as these are obscured by eluting plasma components.

**Fig. 3:**
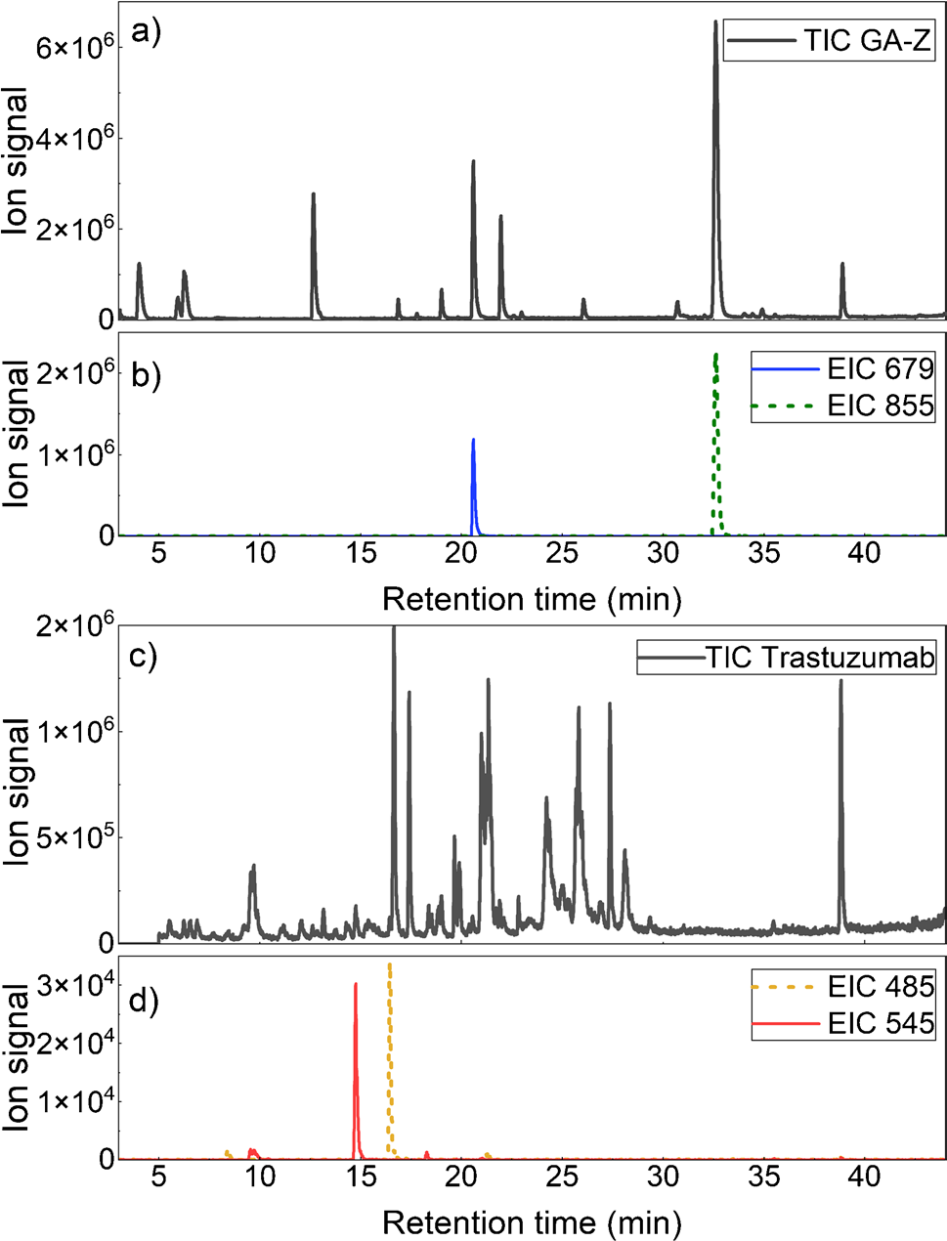
Total ion chromatograms (TIC) of trypsin-digested (**a**) GA-Z and (**c**) trastuzumab, and extraction ion chromatograms (EIC) of the signature peptides of (**b**) GA-Z and (**d**) trastuzumab

Using LC-MS, the two signature peptides, EAANAELDSYGVSDFYKR and TVEGVEALKDAILAALP, were identified for GA-Z. The total ion chromatogram for trypsin-digested GA-Z and the extracted ion chromatograms of the two signature peptides are shown in Fig. 3a and b. For the LC-MS analysis of trastuzumab, the two signature peptides IYPTNGYTR and FTISADTSK from the complementarity-determining region of trastuzumab, identified in earlier studies [34, 35], were used. The signature peptides were also identified with LC-MS in sample with trypsin-digested trastuzumab. The total ion chromatogram for trypsin-digested trastuzumab and the extracted ion chromatograms for the signature peptides are shown in Fig. 3c and d. Further information on both the GA-Z and trastuzumab signature peptides is presented in Table S1 in the Supplementary Information.

**Fig. 4:**
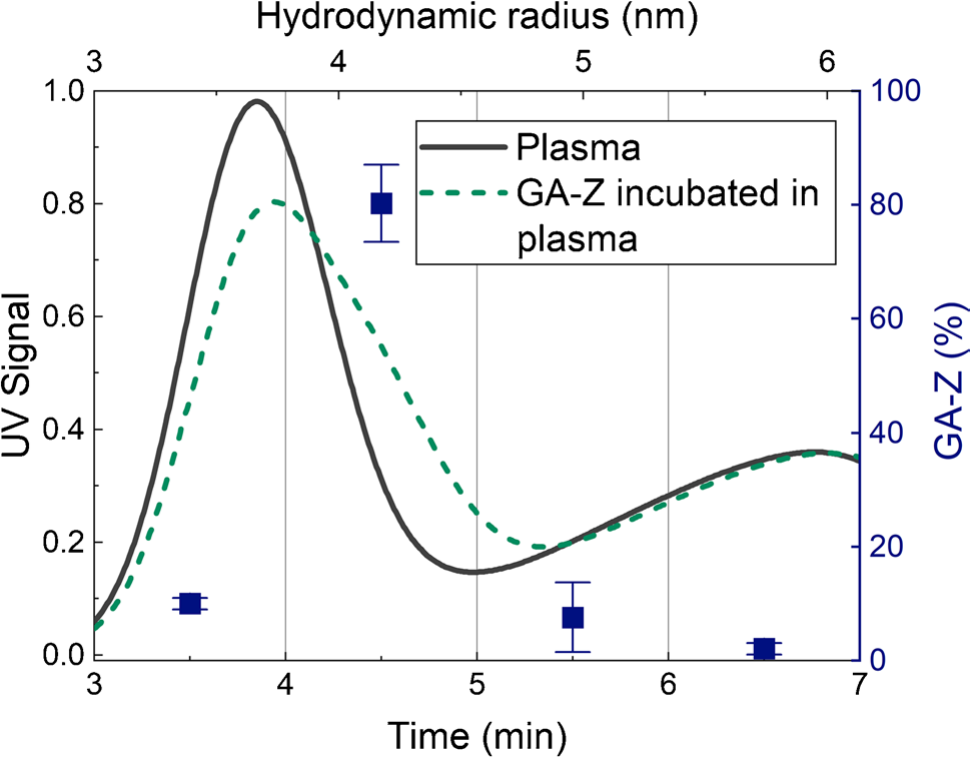
AF4-UV fractogram with mass fractions (blue squares) of the total amount of GA-Z detected in AF4 fractions from separation of GA-Z incubated in plasma. The mass fractions were determined by integrating the extracted ion chromatograms of the GA-Z signature peptides. The integrated data are shown in Table S2 in the Supplementary Information. The standard deviations of the mass fractions are shown by the error bars. All analyses were performed in triplicate

LC-MS analysis of the AF4 fractions shows that the signature peptides of GA-Z were detected in the HSA-dominated peak (Fig. 4). The amount of GA-Z in the trypsin-digested AF4 fractions was determined by integrating the extracted ion chromatograms of the signature peptides. The areas of the integrations are shown in Table S2. Approximately 10% of the total amount of the detected GA-Z eluted at 3–4 min, which corresponds to the first half of the HSA peak. The largest amount of GA-Z, ~80%, eluted at 4–5 min. This corresponds to the second half of the HSA peak, where broadening to longer elution times, i.e., an increase in size, is seen. The results thus show that the binding of GA-Z to HSA can be studied with the methodology presented herein. Lower amounts of GA-Z, 2–8%, were also detected in fractions eluting at 5–6 min and 6–7 min. Its presence in these fractions was most likely caused by the binding of GA-Z to the dimer of HSA (Fig. S2) [45], or the formation of small aggregates, oligomerisation, and/or unspecific binding to larger plasma protein.

**Fig. 5:**
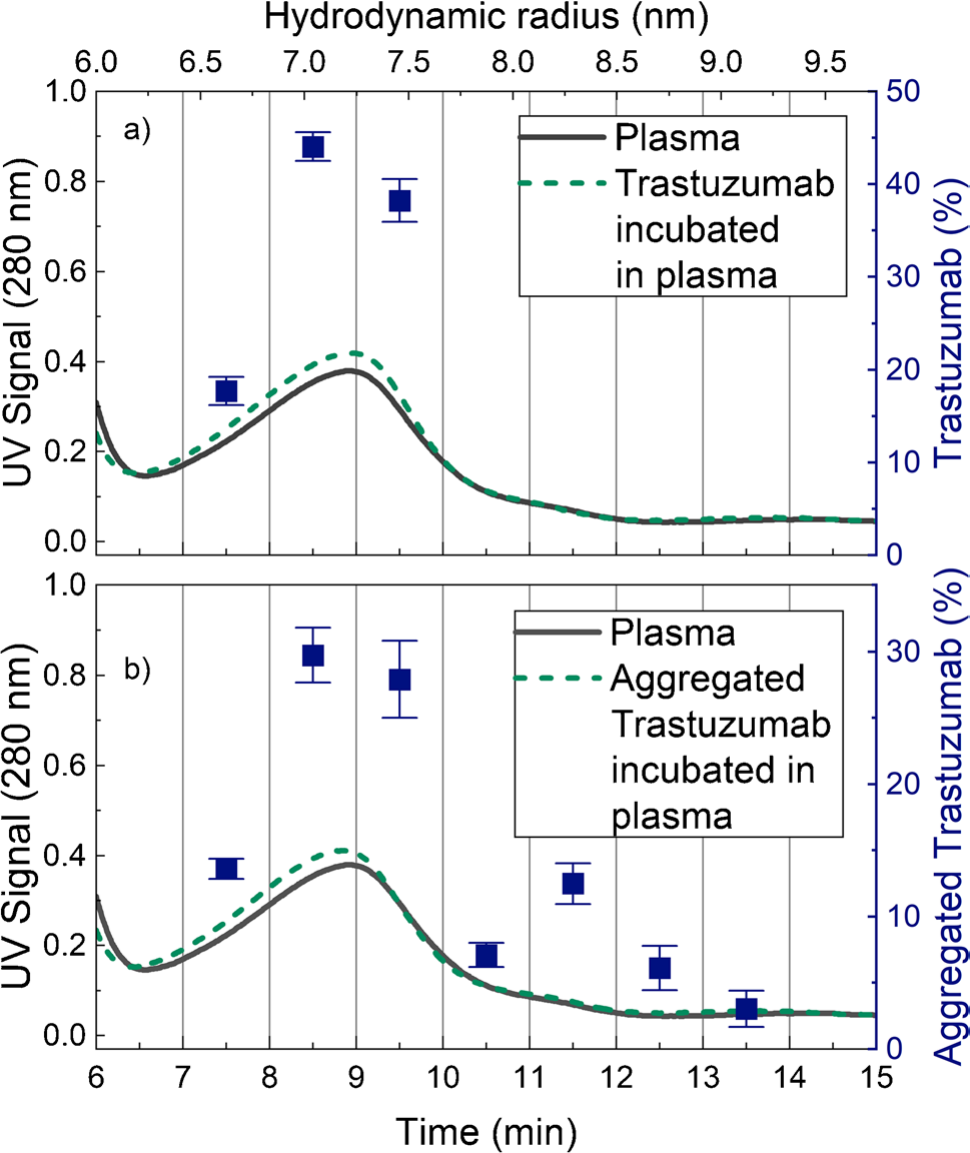
AF4-UV fractograms with mass fractions (blue squares) of the total detected amount of trastuzumab in the eluting fractions. (**a**) Trastuzumab incubated in plasma and (**b**) aggregated trastuzumab incubated in plasma. The mass fractions were determined by integrating the extracted ion chromatograms of the trastuzumab signature peptides. The integrated data are shown in Table S2 in the Supplementary Information. The standard deviations of the mass fractions are shown as error bars. All analyses were performed in triplicate

The signature peptides of native trastuzumab were detected at an elution time of 7–10 min, corresponding to the IgG-dominated peak (Fig. 5). Approximately 18% of the detected amount of trastuzumab eluted at 7–8 min. The majority of trastuzumab eluted at the peak maximum of the IgG-dominated peak, at 8–9 min (~44%) and 9–10 min (~38%). The LC-MS data, therefore, support the observed co-elution of trastuzumab and the IgG peak and show no signs of aggregation or unspecific binding of trastuzumab after 30 min of incubation in plasma. Aggregated trastuzumab was detected in fractions eluting at 7–14 min corresponding to a hydrodynamic radius of ~4.5–9 nm. The amounts of trastuzumab are largest in the fractions corresponding to native trastuzumab, 7–10 min, but have all decreased compared with the mass fraction of trastuzumab after incubation of native trastuzumab. An increase in trastuzumab concentration is observed in the fraction eluting at 11–12 min. The increase correlates well with the elution of the first aggregate peak observed in Fig. 2b, where aggregated trastuzumab is analysed in PBS.

This study has shown that by combining AF4 and LC-MS, the in vitro study of the behaviour of therapeutic proteins in plasma is possible. By combining the separation from AF4, sizes can be determined, while LC-MS yields selective detection and quantitation. The results show that the combination of the techniques allows for the detection of monoclonal antibody aggregates even though the peak of protein aggregates cannot be directly observed due to the complexity of the matrix. Similarly, specific binding to proteins in plasma can also be analysed and characterised. The developed methodology can therefore provide important insights into how therapeutic proteins behave in blood. In addition, it is possible to quantify the amount of aggregates and protein binding in plasma. For absolute quantification, isotope-labelled internal standards of the signature peptides are needed [2].

To extract as much information as possible from the AF4 separation, it is important to lower the detection limit of the protein in the LC-MS analysis as much as possible. Aggregation, oligomerisation, and binding can occur at low levels, therefore generating a low concentration of the proteins in the AF4 fractions. To increase the sensitivity of the LC-MS analysis, sodium deoxycholate was used as the denaturation agent during the tryptic digestion. Studies have shown that the use of sodium deoxycholate instead of urea as the denaturation agent increases the efficiency, peptide recovery, and unbiasedness. For example, it can increase the number of identified proteins and peptides and lower the number of missed cleavages compared with traditional denaturation using urea [46, 47]. The lowest amount of detected trastuzumab in the AF4 fractions, calculated assuming complete recovery, was 34 ng, corresponding to 68 ng/mL and 46 nM. The lowest amount of detected GA-Z, assuming complete recovery, was 19 ng, corresponding to 37 ng/mL and 31 nM. The concentrations of GA-Z and trastuzumab during the incubation in plasma in this study were 0.9 mg/mL. The concentration of therapeutic proteins in the blood may vary between 0.01 ng/mL and 0.1 mg/mL. The concentration of monoclonal antibodies after administration is typically 1–10 mg/mL [2]. Hence, it can also be relevant to incubate the proteins at lower concentrations. To enable the analysis of proteins at lower protein concentrations, it may be necessary to decrease the detection limit of the LC-MS method. This can be achieved by, for instance, further development of the LC-MS method. Improvements could be made, for example, by adding concentration steps and improving the separation of the signature peptides in the LC method and the signal-to-noise ratio of the MS signal by improving the selection and/or ionisation of the signature peptides.

The selected signature peptides of GA-Z and trastuzumab all include asparagine and/or aspartic acid and are therefore susceptible to deamidation and isomerisation. In this study, no deamidation or isomerisation was observed. The proteins were, however, only incubated in plasma for 30 min at room temperature. In the future, it would be of interest to perform studies on the behaviour of chemically degraded proteins in plasma or the deamidation and isomerisation rate ex vivo using the method described in this article together with longer incubation times.

## Conclusion

In this study, a novel in vitro methodology was developed to provide insight into the behaviour of therapeutic proteins in vivo. The method involves a combination of AF4 and LC-MS. The fractionation with AF4 provides valuable information on size which, for example, can indicate binding, oligomerisation, and/or aggregation. The study showed that after incubation of proteins in plasma, this method can be used to investigate and quantify serum albumin binding, analyse changes in monoclonal antibody size (oligomerisation and/or interaction with blood components), and identify and quantify monoclonal antibody aggregates. Thus, this methodological approach opens many possibilities for further studies on the behaviour of therapeutic proteins.

## Supplementary Information

ESM 1 (PDF 410 kb)
