# Supplementary material for: Investigation of native and aggregated therapeutic proteins in human plasma with asymmetrical flow field-flow fractionation and mass spectrometry
Source: Anal Bioanal Chem. 2022 Oct 5;414(29-30):8191–200. doi: 10.1007/s00216-022-04355-2 (PMC9712356; doi:10.1007/s00216-022-04355-2)
Supplement: Supplementary file 1 — (PDF 410 kb) [file 216_2022_4355_MOESM1_ESM.pdf]

Supplementary Information

Analytical and Bioanalytical Chemistry

Investigation of Native and Aggregated Therapeutic Proteins in Human Plasma with Asymmetrical Flow Field-Flow Fractionation and Mass Spectrometry

Ingrid Ramm[*1], Mats Leeman[2], Herje Schagerlöf[3], Ileana Rodríguez León[4], Alejandra Castro[2], Lars Nilsson*[*1]*

[1]*Department of Food Technology, Engineering and Nutrition, Lund University, 221 00 Lund, Sweden*

[2]*SOLVE Research and Consultancy AB, Medicon village, 223 81 Lund, Sweden*

[3]*Department of Chemical Engineering, Lund University, 221 00 Lund, Sweden*

[4]*Analytical Development – Methods and Characterization, Product Development and Drug Delivery, Global Pharmaceutical R&D, Ferring Pharmaceuticals A/S, Amager Strandvej 405, 2770 Kastrup, Denmark*

** Correspondence: ingrid.ramm@food.lth.se; lars.nilsson@food.lth.se*

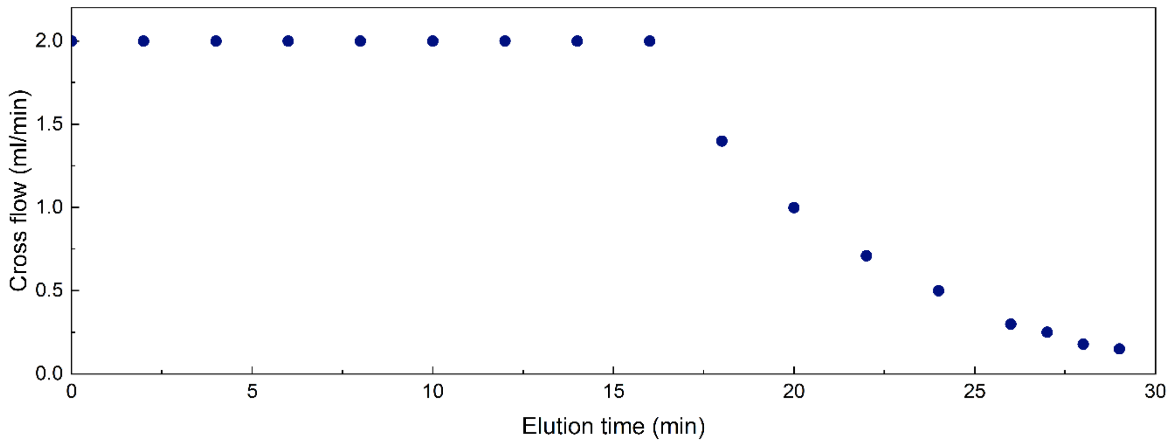

Fig. S1 Crossflow profile of the AF4 separation method. The crossflow was constant at 2 mL/min during 16 min and was then exponentially decreasing to 0.15 mL/min during 16 min

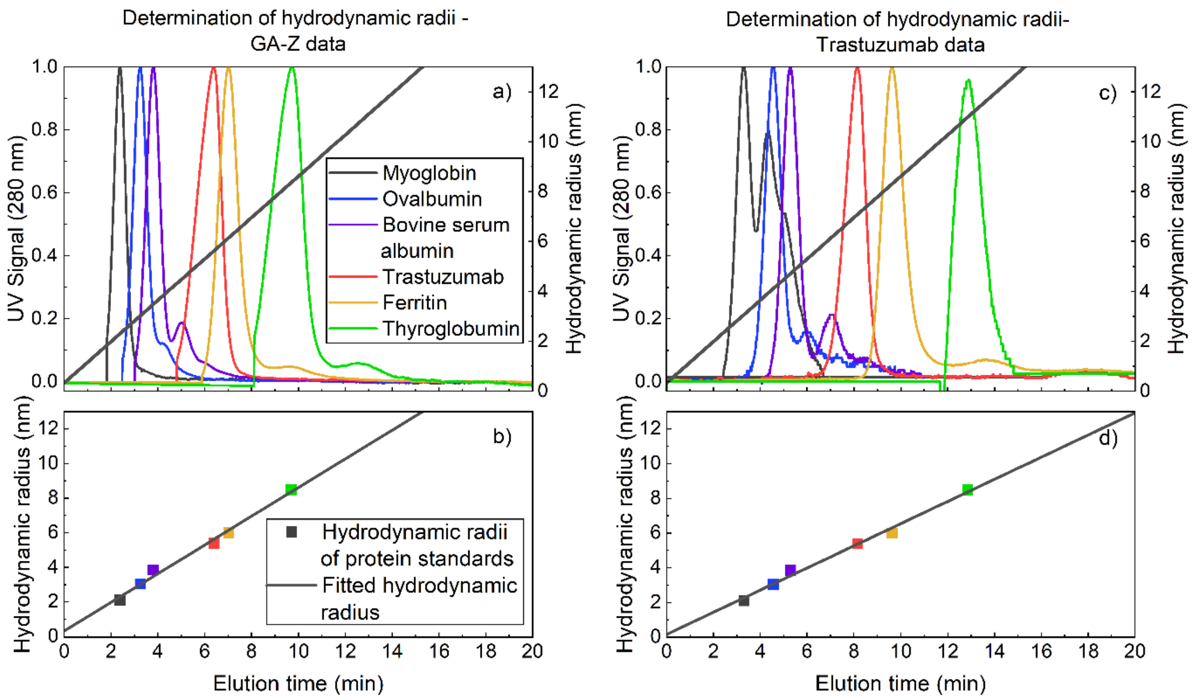

Fig. S2 The hydrodynamic radii of the eluted samples calculated using protein standards. The fractograms of the protein standards in the channel used for separation of samples with a) GA-Z and c) Trastuzumab. Hydrodynamic radii of the protein standard monomers plotted against the elution time of the protein standard monomers in the channel used for the separation of samples with b) GA-Z and d) Trastuzumab. b) and d) also includes fitted lines obtained with linear regression used to determine the hydrodynamic radii in Figure 1-2 and 3-4. The hydrodynamic radii of the protein standard monomers are obtained from literature [1-4]

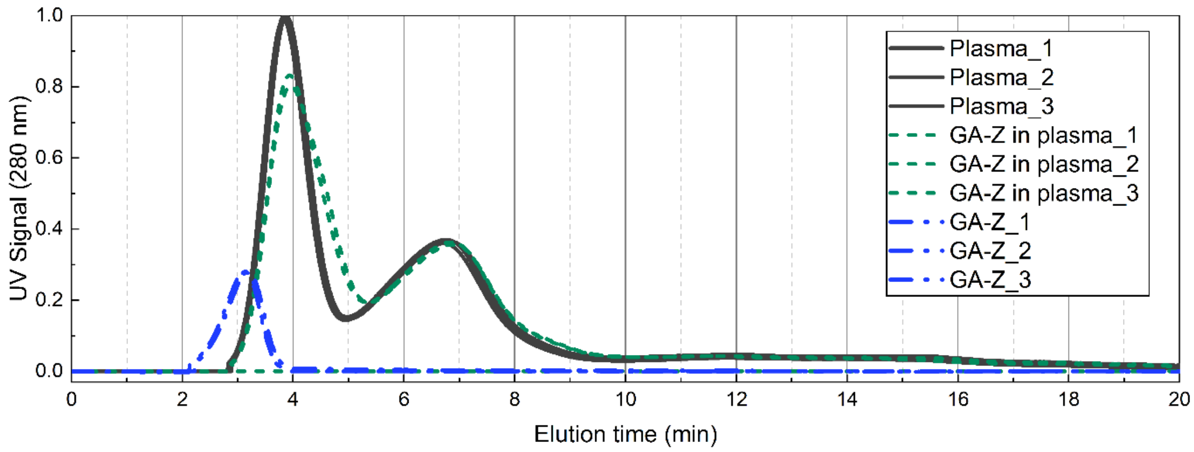

Fig. S3 AF4-UV fractogram of samples with plasma(1-3), native GA-Z(1-3), and GA-Z incubated in plasma(1-3) for 30 min, 20 °C, in PBS buffer

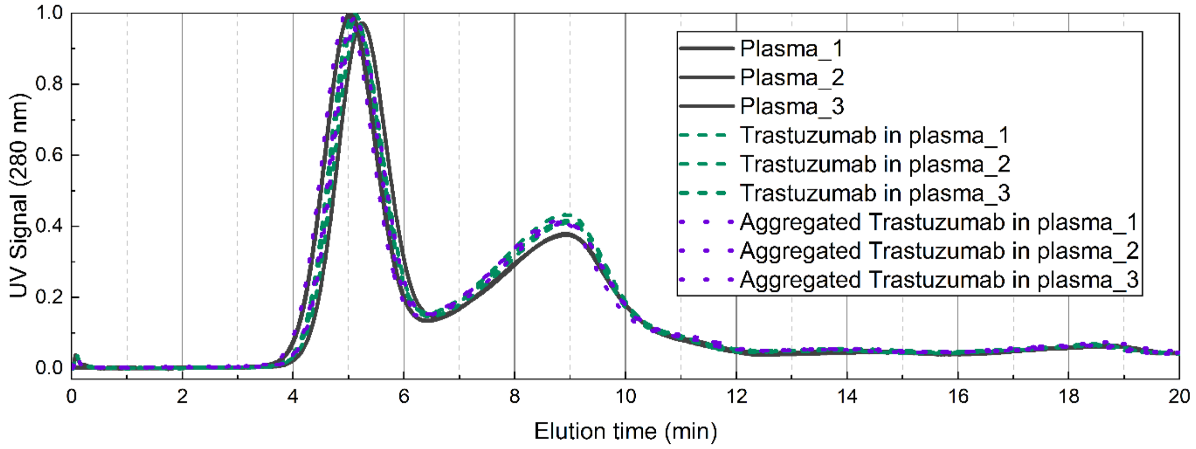

Fig. S4 AF4-UV fractogram of plasma(1-3), Trastuzumab incubated in plasma(1-3), and aggregated Trastuzumab incubated in plasma(1-3), for 30 min, 20 °C, in PBS buffer

Table S1 LC-MS data of GA-Z and Trastuzumab signature peptides

GA-Z signature peptides	Mass (m/z)	Charge state (z)	Retention time (min)	Trastuzumab signature peptides	Mass (m/z)	Charge state (z)	Retention time (Min)
EAANAELDSY GVSDFYKR	679.3	(+3)	20.6	FTISADTSK	485.2	(+2)	16.4
TVEGVEALKD AILAALP	855.5	(+2)	32.6	IYPTNGYTR	543.7	(+2)	14.7

Table S2 LC-MS data of GA-Z, Trastuzumab, and aggregated Trastuzumab incubated in plasma, for 30 min, 20 °C, in PBS buffer and separated with AF4

**Detection of GA-Z incubated in plasma**								
**AF4 Fractions (min)**	**Signature peptide (m/z)**	**Retention time (min)**	**Area**	**%Area**	**Signature peptide (m/z)**	**Retention time (min)**	**Area**	**%Area**
3-4 (1)	679.3	20.4	6520.90	9.290	855.5	32.6	12 112.62	10.036
3-4 (2)	679.3	21.2	17 849.78	8.936	855.5	33.5	15 332.78	9.137
3-4 (3)	679.3	20.7	35 971.95	11.141	855.5	32.8	31 222.18	11.205
Mass fraction in AF4 fraction 3-4 min of the total amount of detected GA-Z: 10.0%±1.0								
4-5 (1)	679.3	20.5	62 155.78	88.546	855.5	32.5	107 775.35	89.273
4-5 (2)	679.3	20.9	148 262.25	74.222	855.5	33.0	128 497.10	76.575
4-5 (3)	679.3	20.7	243 667.40	75.467	855.5	32.8	217 005.70	77.879
Mass fraction in AF4 fraction 4-5 min of the total amount of detected GA-Z: 80.3%±6.8								
**AF4 Fractions (min)**	**Signature peptide (m/z)**	**Retention time (min)**	**Area**	**%Area**	**Signature peptide (m/z)**	**Retention time (min)**	**Area**	**%Area**
5-6 (1)	679.3	0	0	0	855.5	0	0	0
5-6 (2)	679.3	20.8	27 406.47	13.720	855.5	33.0	18 797.15	11.202
5-6 (3)	679.3	20.6	35 757.97	11.075	855.5	32.7	27 552.56	9.888
Mass fraction in AF4 fraction 5-6 min of the total amount of detected GA-Z: 7.6%±6.1								
6-7 (1)	679.3	20.4	1519.36	2.164	855.5	32.5	833.99	0.691
6-7 (2)	679.3	20.8	6236.37	3.122	855.5	33.0	5177.71	3.086
6-7 (3)	679.3	20.7	7481.46	2.317	855.5	35.6	2863.91	1.028
Mass fraction in AF4 fraction 6-7 min of the total amount of detected GA-Z: 2.1%±1.0								
**Detection of Trastuzumab incubated in plasma**								
**AF4 Fractions (min)**	**Signature peptide (m/z)**	**Retention time (min)**	**Area**	**%Area**	**Signature peptide (m/z)**	**Retention time (min)**	**Area**	**%Area**
7-8 (1)	485.2	16.6	34 899.91	16.722	543.7	14.9	27 968.16	18.359
7-8 (2)	485.2	16.3	60 784,58	17.658	543.7	14.6	25 461.42	15.647
7-8 (3)	485.2	16.3	56 241,78	20.126	543.7	14.6	25 742.40	17.801
Mass fraction in AF4 fraction 7-8 min of the total amount of detected Trastuzumab: 17.7%±1.5								
8-9 (1)	485.2	16.6	86 060.93	41.235	543.7	14.9	67 780.12	44.493

8-9 (2)	485.2	16.3	156 326.41	45.413	543.7	14.6	73 658.70	45.267
8-9 (3)	485.2	16.3	124 149.28	44.426	543.7	14.6	62 768.07	43.405
Mass fraction in AF4 fraction 8-9 min of the total amount of detected Trastuzumab: 44.0%±1.6								
9-10 (1)	485.2	16.6	87 747.82	42.043	543.7	14.9	56 590.90	37.148
9-10 (2)	485.2	16.3	127 124.50	36.930	543.7	14.7	63 599.28	39.085
9-10 (3)	485.2	16.3	99 061.35	35.448	543.7	14.6	56 100.29	38.794
Mass fraction in AF4 fraction 9-10 min of the total amount of detected Trastuzumab: 38.5%±2.3								

Detection of aggregated Trastuzumab incubated in plasma

AF4 Fractions (min)	Signature peptide (m/z)	Retention time (min)	Area	%Area	Signature peptide (m/z)	Retention time (min)	Area	%Area
7-8 (1)	485.2	16.3	36 739.38	13.820	543.7	14.7	23 953.07	13.909
7-8 (2)	485.2	16.2	8434.27	12.067	543.7	14.5	8525.00	13.720
7-8 (3)	485.2	16.4	8568.18	13.984	543.7	14.7	7147.59	14.166
Mass fraction in AF4 fraction 7-8 min of the total amount of detected Trastuzumab: 13.6%±0.8								
8-9 (1)	485.2	16.3	82 697.70	31.108	543.7	14.7	48 778.79	28.324
8-9 (2)	485.2	16.3	18 331.01	26.227	543.7	14.6	19 369.84	31.172
8-9 (3)	485.2	16.2	19 343.71	31.570	543.7	14.6	15 123.58	29.974
Mass fraction in AF4 fraction 8-9 min of the total amount of detected Trastuzumab: 29.7%±2.1								
9-10 (1)	485.2	16.3	66 028.54	24.838	543.7	14.7	45 806.02	26.598
9-10 (2)	485.2	16.3	22 782.10	32.595	543.7	14.5	18 037.92	29.029
9-10 (3)	485.2	16.2	15 543.67	25.368	543.7	14.5	14 642.08	29.019
Mass fraction in AF4 fraction 9-10 min of the total amount of detected Trastuzumab: 27.9%±2.9								
10-11(1)	485.2	16.3	20 572.94	7.739	543.7	14.7	12 264.22	7.121
10-11(2)	485.2	16.2	3959.00	5.664	543.7	14.5	4351.21	7.003
10-11(3)	485.2	16.3	4109.58	6.707	543.7	14.6	4200,91	8.326
Mass fraction in AF4 fraction 10-11 min of the total amount of detected Trastuzumab: 7.1%±0.9								
11-12(1)	485.2	16.3	34 998.99	13.166	543.7	14.7	20 084.12	11.662
11-12(2)	485.2	16.2	8510.10	12.176	543.7	14.6	6804.57	10.951
11-12(3)	485.2	16.5	9330.69	15.228	543.7	14.9	5931,68	11.756
Mass fraction in AF4 fraction 11-12 min of the total amount of detected Trastuzumab: 12.5%±1.5								
12-13(1)	485.2	16.3	17 781.81	6.689	543.7	14.6	11 522.20	6.690
12-13(2)	485.2	16.4	6022.79	8.617	543.7	14.7	3685.01	5.930
12-13(3)	485.2	16.5	3203.72	5.229	543.7	14.9	1808.42	3.584
Mass fraction in AF4 fraction 12-13 min of the total amount of detected Trastuzumab: 6.1%±1.7								
13-14(1)	485.2	-	7018.52	2.640	543.7	14.7	9809.76	5.696
13-14(2)	485.2	16.3	1855.36	2.655	543.7	14.7	1364.16	2.195
13-14(3)	485.2	16.5	1172.26	1.913	543.7	14.9	1602.00	3.175
Mass fraction in AF4 fraction 13-14 min of the total amount of detected Trastuzumab: 3.0%±1.4								

1. Wilkins DK, Grimshaw SB, Receveur V, Dobson CM, Jones JA, Smith LJ (1999) Hydrodynamic Radii of Native and Denatured Proteins Measured by Pulse Field Gradient NMR Techniques. Biochemistry 38 (50):16424-16431. doi:10.1021/bi991765q
2. Erickson HP (2009) Size and shape of protein molecules at the nanometer level determined by sedimentation, gel filtration, and electron microscopy. Biol Proced Online 11:32-51. doi:10.1007/s12575-009-9008-x
3. Magnusson E, Hakansson A, Janiak J, Bergenstahl B, Nilsson L (2012) Hydrodynamic radius determination with asymmetrical flow field-flow fractionation using decaying cross-flows. Part II. Experimental evaluation. J Chromatogr A 1253:127-133. doi:10.1016/j.chroma.2012.07.005
4. Espinosa-de la Garza CE, Miranda-Hernández MP, Acosta-Flores L, Pérez NO, Flores-Ortiz LF, Medina-Rivero E (2015) Analysis of therapeutic proteins and peptides using multiangle light scattering coupled to ultra high performance liquid chromatography. Journal of Separation Science 38 (9):1537–1543. doi:10.1002/jssc.201400863
